# The Single-Nucleotide Polymorphism (SNP) Validity to Detect Omicron Variants

**DOI:** 10.1155/2023/6618710

**Published:** 2023-09-07

**Authors:** Lia Gardenia Partakusuma, Luhung Budiailmiawan, Budiman, Ida Parwati, Aryati, Basti Andriyoko, Louisa Markus, Corine Niswara, Cut Nur Cinthia Alamanda

**Affiliations:** ^1^Faculty of Medicine and Post Graduate Programme, YARSI University, Jakarta, West Java, Indonesia; ^2^Palabuhanratu Hospital, Sukabumi, West Java, Indonesia; ^3^COVID Emergency Hospital, Kemayoran, Jakarta, West Java, Indonesia; ^4^Department of Clinical Pathology, Faculty of Medicine Padjadjaran University, Dr. Hasan Sadikin General Hospital, Bandung, West Java, Indonesia; ^5^Department of Clinical Pathology, Faculty of Medicine Airlangga University, Dr. Soetomo General Hospital, Surabaya, East Java, Indonesia; ^6^Cengkareng Hospital, Jakarta, West Jakarta, Indonesia; ^7^West Java Provincial Health Laboratory, Bandung, West Java, Indonesia

## Abstract

**Introduction:**

Mutation of SARS-CoV-2 has generated several variants of concern (VOC) which spread promptly worldwide. These emerging variants affected global strategies to overcome COVID-19. Variants of SARS-CoV-2 are determined by the whole genome sequencing (WGS) assay, which is time-consuming, with limited availability (only in several laboratories). Hence, a faster and more accessible examination is needed. The single-nucleotide polymorphism (SNP) method is one of the options for genomic variation surveillance that can help provide an answer to this challenge. This study aims to determine the validity of the SNP method with PCR to detect omicron variants of SARS-CoV-2 compared with the gold standard, WGS.

**Methods:**

This is a diagnostic analysis of 140 confirmed COVID-19 nasopharyngeal samples taken from the Kemayoran COVID Emergency Hospital Laboratory and the West Java Provincial Health Laboratory from April to October 2022. Data analysis was carried out to determine conformity and validity values.

**Results:**

Analysis using Cohen's kappa coefficient test showed high conformity between SNP and WGS (*p* value <0.001; kappa coefficient = 0.948). SNP showed great validity values on omicron BA.1 (90% sensitivity; 100% specificity), omicron BA.2 (100% sensitivity; 99% specificity), and omicron BA.4/5 (99.2% sensitivity; 100% specificity).

**Conclusion:**

The SNP method can be a more time-efficient alternative to detect omicron variants of SARS-CoV-2 and distinguish their sublineages (BA.1, BA.2, and BA.4/5) by two different specific gene mutations in combination analysis (ΔH69/V70 and Q493R mutations).

## 1. Introduction

The outbreak of a novel coronavirus infection originating from the Hubei province of China caused a worldwide pandemic at the end of 2019 [1]. On February 2020, the World Health Organization (WHO) named this novel coronavirus as severe acute respiratory syndrome coronavirus 2 (SARS-CoV-2) and named the diseases as coronavirus-disease of 2019 (COVID-19). For the past two years, various efforts have been made to overcome the COVID-19 pandemic by conducting strategies regarding examination (testing), isolation, lockdown, and intensive vaccination [2].

Since its first emergence at the end of 2019, SARS-CoV-2 has mutated and generated several variants of concern (VOC) that have rapidly spread globally [3]. On December 29, 2021, there was an increase in COVID-19 cases in the world (10,448,409 cases daily), peaked on January 24, 2022 (23,555,167 cases daily), decreased on April 18, 2022 (4,713,853 cases daily), and then peaked again on July 18, 2022 (7,172,704 cases daily) [4]. The COVID-19 cases in Indonesia experienced a significant increase on June 14, 2021 (78,551 cases daily), peaked on July 12, 2021 (350,273 cases daily), and then experienced a decrease in cases on October 4, 2021 (8,648 cases daily). Then, there was a spike in cases again in February 2022 which peaked on February 14, 2022 [5]. This surge in the number of cases has raised suspicions about a change in the pattern of infection of the SARS-CoV-2 virus variant in Indonesia. The emergence of SARS-CoV-2 variants has affected strategies to overcome COVID-19 [6]. A variant of SARS-CoV-2 is classified as VOC when it has increased transmissibility and/or virulence, and when it has created some changes in disease presentation, diagnostic methods, and management measurement [7]. Variants of SARS-CoV-2 are determined by whole genome sequencing (WGS) examination. Unfortunately, these examinations are carried out only by several laboratories in each region in Indonesia and take approximately 3–5 days. Such limitations give necessity to the need for a faster and easier examination method that can be performed in all molecular laboratories.

The use of quantitative reverse transcription real-time polymerase chain reaction (qRT-PCR) remains the gold standard for testing, wherein unique sequences of the SARS-CoV-2 genome are detected. The diagnostic accuracy of this technique is of utmost importance [8]. The qRT-PCR examination of single-nucleotide polymorphism (SNP) targets is one of the options for genomic variation surveillance that can help bring an answer to this issue. This SNP method can be carried out in all laboratories with biosafety level 2 (BSL-2) because it is PCR-based and can be performed within 2–4 hours with a much faster examination in comparison with WGS [9]. This SNP technique can also predict COVID-19 severity with specific vulnerable gene detection [10].

Omicron is one of the VOCs that spread globally and is regarded as one of the significant public health concerns [11]. Omicron was first identified in South Africa in November 2021. This subvariant SARS-CoV-2 was designated as a VOC on 26^th^ November 2021 [12]. The differences between omicron and the first SARS-CoV-2 genome are as follows: the spike region of the originally BA.1 omicron genome had 35 mutations with 30 amino acid substitutions, three in-frame deletions, and an insertion of three amino acids. Fifteen mutations exist in the receptor-binding domain (RBD), which is the dominant binding site of the virus to host cells and target neutralizing antibodies (Nabs) [11, 13]. Omicron variants also have three and six mutations in the region coding for membrane protein and nucleocapsid protein. Spike protein is the major surface glycoprotein of SARS-CoV-2, which is divided into the N-terminal domain (NTD) and receptor-binding domain (RBD). A small group of 25 amino acids of RBD is responsible for interaction with cellular receptor angiotensin-converting enzyme-2 (ACE-2). Following ACE-2 binding, S1 is cleaved and detached, whereas S2 undergoes a major conformational change to expose the fusion loop, which mediates the fusion of viral and host membranes, allowing viral RNA to enter the host cell cytoplasm and commence the replicative cycle [14]. Some of the mutations in the NTD spike region of omicron have been observed previously in other variants, for example, del69–70 (ΔH69/V70/S-gene test failure/SGTF) in the alpha variant, T951 in kappa and iota variants, and G142D in kappa and delta variants [11].

Substitutions in the RBD of omicron, such as Q493R, N501Y, S371L, S373P, S375F, Q498R, and T478K, have conferred higher binding affinity to ACE-2. The furin cleavage site (FCS), at the junction of S1 and S2, plays a key role in the fusion of the virus with the host cell. Omicron contains 3 substitutions (N679K, H655Y, and P681H) close to the furin cleavage site. 15 RBD and 3 furin cleavage site substitutions in omicron suggest a major change in infectivity [15].

Before the omicron variant, monoclonal antibody therapy has proven to be highly effective in preventing death; however, it may not be as effective for omicron variants [13]. Omicron variants contain mutations within RBD that were previously considered highly conserved and are targets of monoclonal antibodies. Among the 15 RBD substitutions in the omicron variant, the K417N substitution (which is also present in the beta variant) is responsible for the most significant disruption to known mAbs [7]. Some cases of Q493R mutations following bamlanivimab/etesevimab administration were also reported and are associated with reduced viral clearance and causing fatal outcomes for some patients [13, 16]. Several omicron sublineages (BA.1, BA.2, and BA.3) shared the same mutation of Q493R, while BA.2 does not have ΔH69/V70. Omicron lineage BA.4/5 S sequences are identical and closely related to BA.2. Compared to BA.2, BA.4/5 has residues ΔH69/V70, revertant mutant Q493R, and two additional mutations in RBD (L452R and T478K). These two additional mutations are considered the main factors for antibody escape [12, 17]. Based on that, ΔH69/V70 and Q493R mutations are important mutations for omicron to increase its transmissibility and virulence. Therefore, these two mutation sites are used as specific markers for omicron detection.

The omicron variant is reported to have mild COVID-19 disease, even though the omicron variant has high infectivity [18]. The severity of the omicron infection is found to be less than that of the delta variant. The most common symptoms for the omicron variant are coryza, cough, headache, and muscle or limb pain in contrast to the delta variant infection which has common symptoms of smell loss, taste loss, fever, and shortness of breath [19, 20]. Diarrhea, headache, and shortness of breath appear to be the most important symptoms for the lambda variant [21]. Patients infected during the omicron wave were 25% less likely to be admitted to hospital (1.9%) than patients infected during the period of high delta prevalence (2.6%). Patients infected during the omicron wave were also 2.5 times more likely to recover within one week than patients with the delta variant [22]. The previous study found that the delta variant causes long COVID (43%) more common than the omicron variant (8.2%) [23]. Therefore, it is very important to know the variants of SARS-CoV-2.

To handle COVID-19 cases in Indonesia effectively, it is essential to determine the variant type of SARS-CoV-2. Therefore, it is important and necessary to study the validity of SARS-CoV-2 variant determination using the qRT-PCR examination method for SNP. This study aims to determine the validity of single-nucleotide polymorphism (SNP) polymerase chain reaction (PCR) variants of omicron SARS-CoV-2 in comparison with the gold standard whole genome sequencing (WGS).

## 2. Materials and Methods

**Figure 1:**
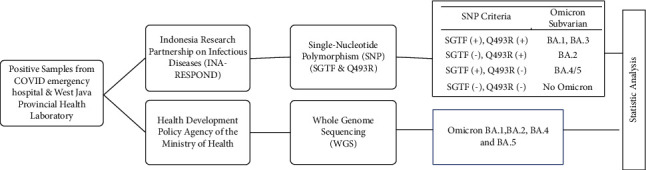
The workflow diagram for SNP and WGS detection.

This is an analytical diagnostic cross-sectional study. Subjects of this study confirmed COVID-19 nasopharyngeal samples were taken from the Laboratory of Kemayoran COVID Emergency Hospital and the West Java Provincial Health Laboratory in the period of April 2022–October 2022. The workflow diagram for SNP and WGS detection is shown in Figure 1.

### 2.1. RNA Extraction

RNAs were extracted using the viral PureLink reagent manual method RNA/DNA kits (Invitrogen, Cat. #12280050). The extraction process uses the manual method of the PURELINK reagent as follows: add 60 mL of 96–100% ethanol to 15 mL of wash buffer and incubate at room temperature. 25 *μ*L of Proteinase K was added into a sterile centrifuge tube of 200 *μ*L specimens, positive control or negative control, and 10 *μ*L internal control. 200 *μ*L lysis buffer (containing 5.6 *μ*g carrier RNA) was added. The tube was closed with a tube cover, homogenized with a vortex for 15 seconds, incubated at 56°C for 15 minutes, and then centrifuged to remove air bubbles. 250 *μ*L of 96–100% ethanol was added to the lysate tube for getting 37% ethanol concentration, vortexed for 15 seconds, incubated for 5 minutes at room temperature, and then centrifuged. The lysate mixed with ethanol was transferred into the spin column and then centrifuged at 6800 × g for 1 minute. The result of the spin column was placed into a clean wash tube (2 mL); then, 500 *μ*L of wash buffer with ethanol was added to the spin column, turning it at a speed of 6800 × g for 1 minute. The lysate mixed with ethanol was transferred into the viral spin column and then centrifuged at 6800 × g for 1 minute. The result was placed into a clean wash tube (2 mL) and then rotated at maximum speed in microcentrifuges for 1 minute. The viral spin column results were placed into a 1.5 mL tube, and then, 10–15 *μ*L of sterile RNAse-free water was added [24, 25].

### 2.2. RT-qPCR Assays

The panel mutation reagent used in this study is the TaqMan panel mutation reagent which has been validated by Neopane et al. [26]. The SNP genotyping examination was carried out based on the TaqMan SARS-CoV-2 mutation panel insert kit (Applied Biosystem, Thermo Fisher Scientific). This assay required 5 *μ*L Taq Path 1-Step RT-qPCR Master Mix, CG; 0.5 *μ*L TaqMan SARS-CoV-2 Mutation Panel Assay; and 9.5 *μ*L nuclease-free water with a total volume of 15 *μ*L of reagent mix in 96 wells and a 0.2 ml plate. The reagent mix was mixed with 5 *μ*L of the sample or nuclease-free water, vortexed for 10–30 seconds, and centrifuged for 1-2 minutes at 650 RCF to remove bubbles. The examination was carried out on QuantStudio 5. The time taken for the whole process of the qRT-PCR examination method of SNP is one hour and ten minutes [24].

The TaqMan SNP genotyping assay consists of a sequence-specific forward and reverse primer that will amplify the sequencing target region. The reverse primers will transcript the SARS-CoV-2 RNA genome sequence. Each test contains two TaqMan minor groove binder (MGB) probes with a nonfluorescent quencher (NFQ) and a 5′ dye reporter (a VIC dye-labeled probe to detect reference sequences and a FAM dye-labeled probe to detect mutation sequences).

**Figure 2:**
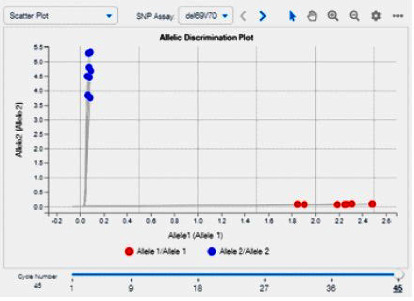
Plot genotyping data from TaqMan SARS-CoV-2 mutation panel assays. Adapted from: TaqMan SARS-CoV-2 mutation panel [27].

The samples containing the reference allele will form clusters on the *X*-axis, and conversely, the samples containing the mutated allele will form clusters on the *Y*-axis [24]. The plot of genotyping data from TaqMan SARS-CoV-2 mutation panel assays is shown in Figure 2.

The target gene mutations used in this study were ΔH69/V70 and Q493R. Omicron sublineages BA.1, BA.2, and BA.3 have the same Q493R mutation, although BA.2 does not have ΔH69/V70. Adversely, omicron sublineages BA.4 and BA.5 have del69–70 but do not have the Q493R mutation [28]. The sample examination was carried out at the Indonesia Research Partnership on Infectious Diseases (INA-RESPOND) Laboratory, Tangerang District Hospital. The single-nucleotide polymorphism (SNP) criteria for omicron sublineages are shown in Table 1.

### 2.3. Data Analysis

**Figure 3:**
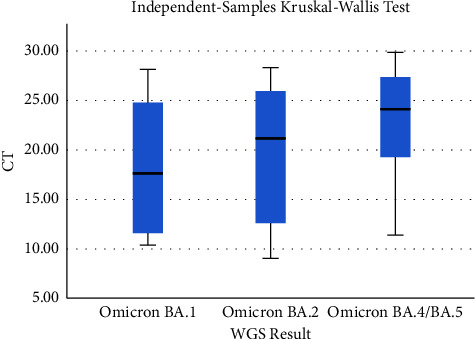
The CT-value distribution between omicron BA.1, BA.2, and BA.4/5.

Statistical analysis is carried out with SPSS version 20, including diagnostic tests (sensitivity, specificity, positive predictive value, and negative predictive value) and conformity test with Cohen's kappa coefficient test. The cycle threshold (CT)-value between Omicron sublineages was compared using the Kruskal–Wallis test is shown in Figure 3.

### 2.4. Ethical Approval

This study was approved by the Ethics Committee for Health Studies at Dr. Hasan Sadikin Hospital, Bandung (LB.02.01/X.6.5/85/2022).

### 2.5. Whole Genome Sequencing

Presence of signature mutations was confirmed by whole genome sequences examined by the Health Development Policy Agency of the Ministry of Health Laboratory from the Laboratory of Kemayoran COVID Emergency Hospital and the West Java Provincial Health Laboratory PCR-positive samples.

## 3. Results and Discussion

A total of 140 nasopharyngeal-positive swab samples were collected from COVID-19 patients who came to Kemayoran COVID Emergency Hospital and the West Java Provincial Health Laboratory in the period of April 2022–October 2022. Diagnostic criteria for SNPs were made based on gene mutations that occur in each sublineage [9]. The characteristics of the subjects are shown in Table 2.

Most of the subjects were females (59.3%) aged 17–33. Most patients experienced mild symptoms (92.1%), with only two experiencing severe symptoms. It is in line with previous studies that indicate omicron symptoms are less severe than delta ones. Moreover, the provision of COVID-19 vaccination makes the symptoms of COVID-19 milder [19]. The WGS result concluded that most of the samples are omicron BA.4/BA.5 (85%), which is in line with the SNP criterion results (84.3%) for omicron BA.4/BA.5. Based on Indonesian GISAID data, omicron BA.1 and BA.2 were still reported in April 2022, while the reports of omicron BA.4 and BA.5 started in May 2022, and the majority of variants were reported up until October 2022 [29].

Cohen's kappa coefficient test to analyze the conformity between the results of SNP and WGS showed high conformity (*p* value <0.001; kappa coefficient 0.948), as shown in Table 3. This result follows a previous study that concluded SNP to have high conformity to WGS and may be useful as an omicron marker, even though validation is required for the given setting [30].

Diagnostic values were determined by sensitivity, specificity, positive predictive value (PPV), negative predictive value (NPV), positive likelihood ratio (LR+), and negative likelihood ratio (LR−) from the SNP examination results in detecting omicron BA.1, BA.2, and BA.4/5 (Table 4). SNP showed good validity to detect omicron BA.1 (99.3% accuracy; 90% sensitivity; 100% specificity), omicron BA.2 (99.3% accuracy; 100% sensitivity; 99% specificity), and omicron BA.4/5 (99.3% accuracy; 99.2% sensitivity; 100% specificity). The results conclude that by combining two different signature mutations in parallel, ΔH69/V70, and Q493R mutations, it is possible to detect the most common omicron sublineages (BA.1, BA.2, and BA.4/5) in circulation. This method enables differentiation between omicron sublineages BA.1 and BA.2 and emerging BA.4/BA.5. Contrary to the research by Jessen et al. (2022), which used ΔH69/V70 and L452R mutations as a target [25], this study used the Q493R mutation as a very specific target for omicron, a major cause of resistance to bamlanivimab/etesevimab as a monoclonal antibody therapy, which is associated with reduced viral clearance and causing fatal outcomes for some patients [13]. This site is essential for monoclonal antibody therapy. But on omicron sublineages BA.4, BA.5, BA.2.75, and BQ.1, XBB produced reverse mutation Q493R to increase ACE-2-S1-RBD affinity, providing some basis for the increased infectivity and severity of the variant [31].

The cycle threshold (CT) value between omicron sublineages was then compared using the Kruskal–Wallis test (Table 5). The median CT value for omicron BA.1 is the lowest (17.7; IQR 11.3–25.0), followed by omicron BA.2 (21.1; IQR 11.7–26.7), and omicron BA.4/5 the highest (24.1; IQR 19.1–27.6). The CT values between the three sublineages show a significant difference (*p* value <0.05) (Figure 1). Mutation Q493R in omicron BA.1 and BA.2 was associated with a rise in SARS-CoV-2 viral load in nasopharyngeal samples; this causes the CT values in BA.1 and BA.2 to be lower than those in other variants. In some cases, the presence of the Q493R mutation was associated with a relapse of COVID-19 with distress respiratory syndrome [13]. Meanwhile, the Q493R reversion mutation in omicron BA.4/BA.5 allows it to regain binding fitness and may even lead to a slightly higher affinity of BA.4/BA.5 for ACE-2 compared to other omicron subvariants. Therefore, symptoms caused by omicron BA.4/BA.5 can be seen in a lower viral load (higher CT-values) than in omicron BA.1 or BA.2 [32].

The limitation of this study is the small number of samples available, especially for omicron sublineages BA.1 and BA.2, which affects the validity of SNP. Smaller samples than necessary would have insufficient statistical power to answer the primary research question, and a statistically nonsignificant result could merely be because of inadequate sample size. The results of this study may only apply to the population under study; they cannot be generalized to other populations, especially to the omicron sublineages BA.1 and BA.2 [33]. This study can only detect and differentiate between omicron BA.1, BA.2, and BA.4/5. The study could not distinguish BA.4 from BA.5 and could not detect other omicron sublineages. Therefore, additional specific SNP mutation targets are required to detect and differentiate other omicron sublineages.

## 4. Conclusions

The use of the SNP method to determine omicron variants has produced a good validity value that conforms to WGS, which is considered the gold standard. Taking into account the efficiency of time and facility requirements, the SNP method can be used as an alternative assay to detect omicron variants. Combined with the analysis of two different specific gene mutations (ΔH69/V70 and Q493R mutations), it is possible to detect omicron sublineages (BA.1, BA.2, and BA.4/5).

### 4.1. Suggestions

A further study with a larger number of samples of omicron, especially sublineages BA.1 and BA.2, is recommended to increase statistical power. It is important and necessary to determine specific mutation targets for SNP to differentiate sublineages and severity of different omicron variants in accordance with the ongoing situation of the pandemic.

## Figures and Tables

**Table 1:** SNP criteria for omicron.

SNP criteria	Analysis
SGTF positive, Q493R positive	Omicron BA.1 and omicron BA.3
SGTF negative, Q493R positive	Omicron BA.2
SGTF positive, Q493R negative	Omicron BA.4/5
SGTF negative, Q493R negative	No omicron

**Table 2:** Characteristics of the subjects.

Characteristics	Subjects (*N* = 140) *N* (%)
Gender	
Male	57 (40.7)
Female	83 (59.3)
Age (years old)	
0–16	20 (14.3)
17–33	70 (50.0)
34–50	29 (20.7)
51–67	16 (11.4)
≥68	5 (3.6)
Symptom	
Asymptomatic	7 (5.0)
Mild symptom	129 (92.1)
Moderate symptom	2 (1.4)
Severe symptom	2 (1.4)
SNP genotyping Q493R	
Positive	21 (15.0)
Negative	119 (85.0)
S-gene target failure (SGTF)	
Positive	127 (90.7)
Negative	13 (9.3)
SNP criterion results	
No omicron (negative SGTF, negative Q493)	1 (0.7)
Omicron BA.1 (positive SGTF, positive Q493R)	9 (6.4)
Omicron BA.2 (negative SGTF, positive Q493R)	12 (8.6)
Omicron BA.4/5 (positive SGTF, negative Q493R)	118 (84.3)
WGS criterion results	
Omicron BA.1	10 (7.1)
Omicron BA.2	11 (7.9)
Omicron BA.4/5	119 (85.0)

**Table 3:** Conformity of SNP and WGS results.

SNP results	WGS results			*p* value	Kappa coefficient
	Omicron BA.1	Omicron BA.2	Omicron BA.4/5		
Omicron BA.1	9	0	0	<0.001^*∗*^	0.948
Omicron BA.2	1	11	0		
Omicron BA.4/5	0	0	118		
No omicron	0	0	1		

**Table 4:** Diagnostic values of SNP towards WGS.

SNP criterion results	Diagnostic value						
	Accuracy	Sens	Spec	PPV	NPV	LR+	LR−
Omicron BA.1	99.3	90.0	100.0	100.0	99.2	—	0.1
Omicron BA.2	99.3	100.0	99.2	91.7	100.0	129	0.0
Omicron BA.4/5	99.3	99.2	100.0	100.0	95.5	—	0.01

**Table 5:** The difference between CT values and WGS results.

	WGS results			*p* value^*∗*^
	Omicron BA.1 *n* = 10	Omicron BA.2 *n* = 11	Omicron BA.4/5 *n* = 119	
CT value				
Median (IQR)	17.7 (11.3–25.0)	21.2 (11.7–26.7)	24.1 (19.1–27.6)	0.015^*∗*^
Min-max	10.4–28.1	8.9–28.4	11.4–29.8	

^*∗*^Kruskal–Wallis test.

## Data Availability

The research data are available in supplementary materials.

## Abbreviations

COVID-19: Coronavirus disease
LR+: Positive likelihood ratio
LR−: Negative likelihood ratio
NPV: Negative predictive value
PPV: Positive predictive value
PDS PatKLin: The Indonesian Association of Clinical Pathology and Medical Laboratory
RT-PCR: Reverse transcriptase polymerase chain reaction
SNP: Single-nucleotide polymorphism
SARS-CoV-2: Severe acute respiratory syndrome coronavirus 2
SGTF: S-gene test failure
VOC: Variants of concern
WGS: Whole genome sequencing
WHO: World Health Organization.

## Supplementary Materials

Supplementary Materials All whole genome sequencing and single-nucleotide polymorphism data from patients.
