# Supplementary material for: The Single-Nucleotide Polymorphism (SNP) Validity to Detect Omicron Variants
Source: Adv Virol. 2023 Sep 7;2023:6618710. doi: 10.1155/2023/6618710 (PMC10501844; doi:10.1155/2023/6618710)
Supplement: Supplementary Materials — All whole genome sequencing and single-nucleotide polymorphism data from patients. [file 6618710.f1.zip › Ethical Approval Luhung Budiailmiawan, dr., SpPK(K).pdf]

DATA PENELITI

NO. REGISTRASI	:	03.22.85
NO. PERSETUJUAN ETIK	:	LB.02.01/X.6.5/85/2022
NAMA PENELITI UTAMA	:	Luhung Budiailmiawan, dr.,SpPK(K)
NPM/NIP/NIDK	:	198004042009021003
JUDUL PENELITIAN	:	Uji Diagnostik Pemeriksaan *Polymerase Chain Reaction* (PCR) SARS-CoV-2 Varian Omicron Menggunakan *Single Nucleotide Polymorphisms* (SNP) Terhadap *Whole Genome Sequencing* (WGS) Sebagai Baku Emas
INSTITUSI PENGUSUL	:	Perhimpunan Dokter Spesialis Patologi Klinik dan Kedokteran Laboratorium Indonesia (PDS PatKLin)
JENJANG / KLASIFIKASI	:	13
JENIS PENELITIAN	:	■ Uji Klinis □ Non Uji Klinis
TANGGAL MENDAFTAR	:	16 Desember 2021
PERPANJANGAN / AMANDEMEN		-
TANGGAL VERIFIKASI	:	14 Februari 2022
KEPUTUSAN VERIFIKASI	:	□ Disetujui<br>■ Disetujui dengan perbaikan kecil<br>□ Perlu Perombakan mendasar pada protokol<br>□ Ditolak
TANGGAL TERIMA PERBAIKAN	:	
KEPUTUSAN AKHIR	:	**DISETUJUI**
TANGGAL TANDA TANGAN	:	15 Maret 2022
KOTA PENANDATANGANAN	:	Bandung
NAMA YANG BERTANDA TANGAN	:	Dr. Ina Rosalina, dr., Sp.A(K)., M.Kes., MH.Kes.
SEKRETARIS/SEKRETARIAT	:	■ NNH ■ RZL ■ AHH

KEMENTERIAN KESEHATAN REPUBLIK INDONESIA
DIREKTORAT JENDERAL PELAYANAN KESEHATAN
RSUP Dr. HASAN SADIKIN BANDUNG

Jalan Pasteur No. 38, Bandung 40161

Telepon : (022) 2034953, 2034954 (*hunting*) Faksimile : (022) 2032216, 2032533

Laman : www.rshs.or.id Pos-el : rsup@rshs.web.id

SMS hotline : 08112335555, *Contact Center* : 022 – 2551111, Reservasi *Online* : reservasi.rshs.or.id, *facebook* : /rshsbdg, *twitter*@rshsbdg

No. Reg. 03.22.85

PERSETUJUAN ETIK
ETHICAL APPROVAL
NOMOR : LB.02.01/X.6.5/85/2022

Komite Etik Penelitian RSUP Dr. Hasan Sadikin Bandung, dalam upaya melindungi hak asasi dan kesejahteraan subjek penelitian serta menjamin bahwa penelitian yang menggunakan formulir survei/registrasi/surveilens/Bahan Biologi Tersimpan dan non klinis lainnya berjalan dengan memperhatikan implikasi etik, sosial dan non klinis lainnya yang berlaku, telah mengkaji dengan teliti proposal penelitian berjudul :

The Research Ethics Committee of Dr. Hasan Sadikin General Hospital Bandung, in order to protect the right and welfare of the research subject and to guaranty that the research using survey questionnaire/registry/surveillance/archived biological materials/other non vlinical materials, will carried out according to ethical, legal social implications and other applicable regulations, has been throughtly reviewed the proposal entitled:

"Uji Diagnostik Pemeriksaan *Polymerase Chain Reaction* (PCR) SARS-CoV-2 Varian Omicron Menggunakan *Single Nucleotide Polymorphisms* (SNP) Terhadap *Whole Genome Sequencing* (WGS) Sebagai Baku Emas"

Nama Peneliti Utama<br>*Principal Reseacher*	:	Luhung Budiailmiawan, dr.,SpPK(K)
Pembimbing/Peneliti Lain<br>*Supervisior/Other Researcher*	:	Basti Andriyoko, dr., SpPK(K)<br>Louisa Markus, dr., SpPK<br>Prof. Dr. dr. Aryati, MS, SpPK(K)<br>Prof. Dr. dr. Ida Parwati, SpPK(K).PhD<br>Dr. dr. Lia Gardenia Partakusuma, SpPK(K), MM, MARS
Nama Institusi<br>*Institution*	:	Perhimpunan Dokter Spesialis Patologi Klinik dan Kedokteran Laboratorium Indonesia (PDS PatKLin)

proposal tersebut dapat disetujui pelaksanaannya.
hereby declare that the proposal approved

a. **Penelitian dapat dilaksanakan setelah mendapatkan Surat Izin Penelitian dari direktur atau pejabat terkait RSUP Dr. Hasan Sadikin Bandung atau institusi dimana penelitian dilaksanakan.**
b. Pada akhir penelitian laporan pelaksanaan penelitian harus diserahkan kepada Komite Etik Penelitian RSUP Dr. Hasan Sadikin Bandung.
Jika ada perubahan protokol dan/atau perpanjangan penelitian, harus mengajukan Kembali permohonan kajian etik penelitian (amandemen protokol).

a. ***Research can be carried out after obtaining a research permit from director or related officials Dr. Hasan Sadikin General Hospital Bandung or the institution where the research is carried out.***
b. At the end of research, a report of reseach implementation must be submitted to Health Research Ethics Committee of Dr. Hasan Sadikin General Hospital Bandung.
If there's a change of protocol and/or a research extension, the principal investigator is required to resubmit the protocol for approval (protocol amandment).

Ditetapkan di : Bandung
Issued in
Tanggal : 15 Maret 2022
Date

Ketua
Chairman,

Dr. Ina Rosalina, dr., Sp.A(K)., M.Kes., MH.Kes.
NIP. 196010251987032001

Ethical approval **berlaku satu tahun dari tanggal persetujuan**
**Peneliti berkewajiban :
1. Menjaga kerahasiaan identitas subjek penelitian
Keep the subject's confidential
2. Memberitahukan status penelitian apabila :
   a. Setelah masa berlakunya keterangan lolos kaji etik, penelitian masih belum selesai, dalam hal ini *ethical approval* dan surat izin penelitian harus diperpanjang
   b. Penelitian berhenti ditengah jalan
   Notify of the research status:
   a. There after of period of passing Ethical Approval, a research still not completed, this matter ethical approval and research permit have to be extended
   b. Study ended midway
3. Melaporkan kejadian serius yang tidak diinginkan (*serious adverse events*)
Report a undersirable serious event (serious adverse events)
4. Melaporkan pelaksanaan penelitian secara berkala
Report a study on periodically
