# Supplementary material for: The Single-Nucleotide Polymorphism (SNP) Validity to Detect Omicron Variants
Source: Adv Virol. 2023 Sep 7;2023:6618710. doi: 10.1155/2023/6618710 (PMC10501844; doi:10.1155/2023/6618710)
Supplement: Supplementary Materials — All whole genome sequencing and single-nucleotide polymorphism data from patients. [file 6618710.f1.zip › Research Data.pdf]

No	Sample	WGS Results	Sex	CT	Age	SNP Genotyping result	SGTF
1	SNP01	Omicron BA.5.2	Perempuan	29.8	32	Negatif Q493R	Pos
2	SNP02	Omicron BA.5.2	Perempuan	27.9	43	Negatif Q493R	Pos
3	SNP04	Omicron BA.5.2.1	Laki-laki	16.0	31	Negatif Q493R	Pos
4	SNP05	Omicron BA.5.2.1	Perempuan	27.3	19	Negatif Q493R	Pos
5	SNP06	Omicron BA.5.2.1	Perempuan	22.4	26	Negatif Q493R	Pos
6	SNP07	Omicron BA.5.6	Perempuan	26.8	36	Negatif Q493R	Pos
7	SNP08	Omicron BA.5.2	Perempuan	25.4	70	Negatif Q493R	Pos
8	SNP09	Omicron BA.5.2	Laki-laki	26.8	71	Negatif Q493R	Pos
9	SNP010	Omicron BA.5.2	Laki-laki	26.3	44	Negatif Q493R	Pos
10	SNP011	Omicron BA.5.2	Perempuan	28.3	61	Negatif Q493R	Pos
11	SNP012	Omicron BA.5.2	Perempuan	26.1	25	Negatif Q493R	Pos
12	SNP013	Omicron BA.5.2	Perempuan	28.7	23	Negatif Q493R	Pos
13	SNP014	Omicron BA.5.2.1	Perempuan	13.4	63	Negatif Q493R	Pos
14	SNP015	Omicron BA.5.2.1	Perempuan	13.5	66	Negatif Q493R	Pos
15	SNP016	Omicron BA.5.2	Perempuan	15.7	46	Negatif Q493R	Pos
16	SNP017	Omicron BA.5.2.1	Laki-laki	20.1	64	Negatif Q493R	Pos
17	SNP018	Omicron BA.5	Perempuan	26.8	1	Negatif Q493R	Pos
18	SNP019	Omicron BA.5.1	Perempuan	28.1	25	Negatif Q493R	Pos
19	SNP020	Omicron BA.5.2	Laki-laki	29.2	2	Negatif Q493R	Pos
20	SNP021	Omicron BA.5.2.1	Laki-laki	16.1	2	Negatif Q493R	Pos
21	SNP022	Omicron BA.5.2.1	Perempuan	20.0	55	Negatif Q493R	Pos
22	SNP023	Omicron BA.5.2	Perempuan	17.3	58	Negatif Q493R	Pos
23	SNP024	Omicron BA.5.2	Perempuan	17.7	12	Negatif Q493R	Pos
24	SNP025	Omicron BA.5.2	Perempuan	24.6	41	Negatif Q493R	Pos
25	SNP026	Omicron BA.5.2	Perempuan	26.8	47	Negatif Q493R	Pos
26	SNP027	Omicron BA.5.2.3	Perempuan	26.8	23	Negatif Q493R	Pos
27	SNP028	Omicron BA.5.2	Perempuan	28	23	Negatif Q493R	Pos
28	SNP029	Omicron BA.5.2	Laki-laki	28.6	31	Negatif Q493R	Pos
29	SNP030	Omicron BA.5.2	Perempuan	29.4	23	Negatif Q493R	Pos
30	SNP031	Omicron BA.5.2	Perempuan	22.2	26	Negatif Q493R	Pos
31	SNP032	Omicron BA.5.2.1	Perempuan	19.9	23	Negatif Q493R	Pos
32	SNP036	Omicron BA.5	Laki-laki	21.8	2 bln	Negatif Q493R	Pos
33	SNP037	Omicron BA.5	Perempuan	28.2	2	Negatif Q493R	Pos
34	SNP040	Omicron BA.5	Laki-laki	24.5	17	Negatif Q493R	Pos
35	SNP041	Omicron BA.5	Perempuan	26.7	55	Negatif Q493R	Pos
36	SNP043	Omicron BA.5	Perempuan	14.8	68	Negatif Q493R	Pos
37	SNP044	Omicron BA.5	Perempuan	17.1	15	Negatif Q493R	Pos
38	SNP047	Omicron BA.5	Perempuan	29.4	48	Negatif Q493R	Pos
39	SNP048	Omicron BA.5	Laki-laki	15.1	38	Negatif Q493R	Pos
40	SNP051	Omicron BA.5	Perempuan	25.6	10	Negatif Q493R	Pos
41	SNP052	Omicron BA.5	Laki-laki	27,9	43	Negatif Q493R	Pos
42	SNP053	Omicron BA.5	Perempuan	28.2	47	Negatif Q493R	Pos
43	SNP054	Omicron BA.5	Perempuan	29	8	Negatif Q493R	Pos
44	SNP055	Omicron BA.5	Perempuan	20	28	Negatif Q493R	Pos
45	SNP057	Omicron BA.5	Perempuan	25.5	33	Negatif Q493R	Pos
46	SNP058	Omicron BA.5	Perempuan	27.2	51	Negatif Q493R	Pos

47	SNP059	Omicron BA.5	Perempuan	27.7	24	Negatif Q493R	Pos
48	SNP060	Omicron BA.5	Laki-laki	29.2	6	Negatif Q493R	Pos
49	SNP061	Omicron BA.5	Perempuan	29.4	43	Negatif Q493R	Pos
50	SNP062	Omicron BA.5	Laki-laki	29.5	37	Negatif Q493R	Pos
51	SNP063	Omicron BA.5	Laki-laki	29.6	32	Negatif Q493R	Pos
52	SNP064	Omicron BA.5	Laki-laki	27.3	24	Negatif Q493R	Pos
53	SNP065	Omicron BA.5	Perempuan	16.1	56	Negatif Q493R	Pos
54	SNP067	Omicron BA.5	Laki-laki	20.7	16	Negatif Q493R	Pos
55	SNP068	Omicron BA.5	Perempuan	25.3	36	Negatif Q493R	Pos
56	SNP069	Omicron BA.5	Laki-laki	26.0	19	Negatif Q493R	Pos
57	SNP071	Omicron BA.5	Perempuan	28.6	42	Negatif Q493R	Pos
58	SNP072	Omicron BA.5	Perempuan	27.2	30	Negatif Q493R	Pos
59	SNP073	Omicron BA.5	Perempuan	28.7	30	Negatif Q493R	Pos
60	SNP076	Omicron BA.5	Laki-laki	25.6	68	Negatif Q493R	Pos
61	SNP077	Omicron BA.5	Laki-laki	17.9	31	Negatif Q493R	Pos
62	SNP078	Omicron BA.5	Perempuan	18.7	21	Negatif Q493R	Pos
63	SNP079	Omicron BA.5	Perempuan	23.8	21	Negatif Q493R	Pos
64	SNP080	Omicron BA.5	Perempuan	19.1	25	Negatif Q493R	Pos
65	SNP081	Omicron BA.5	Laki-laki	28.3	24	Negatif Q493R	Pos
66	SNP082	Omicron BA.5	Perempuan	20.3	38	Negatif Q493R	Pos
67	SNP083	Omicron BA.5	Laki-laki	14.7	74	Negatif Q493R	Pos
68	SNP084	Omicron BA.5	Laki-laki	16.5	5 bln	Negatif Q493R	Pos
69	SNP088	Omicron BA.5.2	Laki-laki	28.2	23	Negatif Q493R	Pos
70	SNP089	Omicron BA.5	Perempuan	17.1	25	Negatif Q493R	Pos
71	SNP090	Omicron BA.5.2	Laki-laki	26.6	22	Negatif Q493R	Pos
72	SNP091	Omicron BA.5.2	Laki-laki	24.1	39	Negatif Q493R	Pos
73	SNP092	Omicron BA.5	Laki-laki	18.6	27	Negatif Q493R	Pos
74	SNP094	Omicron BA.5.2	Laki-laki	22.1	13	Negatif Q493R	Pos
75	SNP095	Omicron BA.5.2	Perempuan	12.1	24	Negatif Q493R	Pos
76	SNP096	Omicron BA.5.2	Laki-laki	15.8	28	Negatif Q493R	Pos
77	SNP097	Omicron BA.5.2	Perempuan	23.9	24	Negatif Q493R	Pos
78	SNP098	Omicron BA.5.2	Laki-laki	26.3	31	Negatif Q493R	Pos
79	SNP099	Omicron BA.5.2	Perempuan	25.7	38	Negatif Q493R	Pos
80	SNP100	Omicron BA.5.1	Laki-laki	20.2	15	Negatif Q493R	Pos
81	SNP101	Omicron BA.5.2	Perempuan	23.6	16	Negatif Q493R	Pos
82	SNP102	Omicron BA.5.2	Perempuan	25.5	62	Negatif Q493R	Pos
83	SNP103	Omicron BA.5.2	Laki-laki	29.3	30	Negatif Q493R	Pos
84	SNP104	Omicron BA.5.2	Perempuan	21.1	67	Negatif Q493R	Pos
85	SNP105	Omicron BA.5.2	Laki-laki	21	22	Negatif Q493R	Pos
86	SNP106	Omicron BA.5.6	Perempuan	21.9	31	Negatif Q493R	Pos
87	SNP108	Omicron BA.5.2	Perempuan	22.1	44	Negatif Q493R	Pos
88	SNP109	Omicron BA.5.2	Perempuan	22.2	21	Negatif Q493R	Pos
89	SNP111	Omicron BA.5.2	Perempuan	17.2	6 bln	Negatif Q493R	Pos
90	SNP113	Omicron BA.5.2	Laki-laki	21.4	34	Negatif Q493R	Pos
91	SNP114	Omicron BA.5.2	Perempuan	14.4	60	Negatif Q493R	Pos
92	SNP115	Omicron BA.5.2.1	Perempuan	20.6	26	Negatif Q493R	Pos
93	SNP116	Omicron BA.5.2.1	Perempuan	15.4	23	Negatif Q493R	Pos

94	SNP117	Omicron BA.5.2	Perempuan	18.1	64	Negatif Q493R	Pos
95	SNP118	Omicron BA.5.2	Laki-laki	24.2	45	Negatif Q493R	Pos
96	SNP119	Omicron BA.5.2	Perempuan	28.9	29	Negatif Q493R	Pos
97	SNP120	Omicron BA.5.2	Perempuan	12.6	19	Negatif Q493R	Pos
98	SNP121	Omicron BA.5.2.1	Laki-laki	11.4	21	Negatif Q493R	Pos
99	SNP122	Omicron BA.5.2	Perempuan	13,8	18	Negatif Q493R	Pos
100	SNP123	Omicron BA.5.2	Perempuan	19.3	18	Negatif Q493R	Pos
101	SNP124	Omicron BA.5.2	Perempuan	17.4	41	Negatif Q493R	Pos
102	SNP125	Omicron BA.5.2	Perempuan	22.5	15	Negatif Q493R	Pos
103	SNP126	Omicron BA.5.2	Perempuan	28.5	42	Negatif Q493R	Pos
104	SNP127	Omicron BA.5.2.1	Perempuan	23.2	27	Negatif Q493R	Pos
105	SNP128	Omicron BA.5.2	Perempuan	27.7	22	Negatif Q493R	Pos
106	SNP129	Omicron BA.5.2	Perempuan	26.2	27	Negatif Q493R	Pos
107	SNP131	Omicron BA.5.2	Perempuan	27.6	8	Negatif Q493R	Pos
108	SNP132	Omicron BA.5	Laki-laki	17.0	56	Negatif Q493R	Pos
109	SNP133	Omicron BA.5.2	Perempuan	28.2	48	Negatif Q493R	Pos
110	SNP135	Omicron BA.5.2	Perempuan	17.1	10	Negatif Q493R	Pos
111	SNP136	Omicron BA.5.2.5	Laki-laki	22.3	29	Negatif Q493R	Pos
112	SNP137	Omicron BA.5.2	Laki-laki	23	20	Negatif Q493R	Pos
113	SNP138	Omicron BA.5.2	Laki-laki	25.2	21	Negatif Q493R	Pos
114	SNP139	Omicron BA.5.2	Laki-laki	21.0	19	Negatif Q493R	Pos
115	SNP140	Omicron BA.5.2	Laki-laki	25.6	20	Negatif Q493R	Pos
116	SNP141	Omicron BA.5.2	Laki-laki	28.3	21	Negatif Q493R	Pos
117	SNP142	Omicron BA.5.2.1	Laki-laki	26.4	19	Negatif Q493R	Pos
118	SNP03	Omicron BA.4	Perempuan	21.2	52	Negatif Q493R	Neg
119	SNP066	Omicron BA.4	Laki-laki	19.7	41	Negatif Q493R	Pos
120	SNP143	Omicron BA.1	Perempuan	19.09	26	Positif Q493R	Pos
121	SNP144	Omicron BA.1	Laki-laki	18.03	38	Positif Q493R	Pos
122	SNP145	Omicron BA.1	Perempuan	17.3	44	Positif Q493R	Pos
123	SNP146	Omicron BA.1	Perempuan	11.5	55	Positif Q493R	Pos
124	SNP147	Omicron BA.1	Laki-laki	25.47	32	Positif Q493R	Pos
125	SNP148	Omicron BA.1	Laki-laki	10.8	41	Positif Q493R	Pos
126	SNP149	Omicron BA.1	Perempuan	12.5	35	Positif Q493R	Pos
127	SNP150	Omicron BA.1	Laki-laki	10.4	51	Positif Q493R	Pos
128	SNP151	Omicron BA.1	Laki-laki	24.8	29	Positif Q493R	Pos
129	SNP152	Omicron BA.1	Perempuan	28.11	52	Positif Q493R	Neg
130	SNP153	Omicron BA.2	Perempuan	13.30	27	Positif Q493R	Neg
131	SNP154	Omicron BA.2	Laki-laki	28.35	23	Positif Q493R	Neg
132	SNP155	Omicron BA.2	Laki-laki	19.14	48	Positif Q493R	Neg
133	SNP156	Omicron BA.2	Laki-laki	11.68	31	Positif Q493R	Neg
134	SNP157	Omicron BA.2	Laki-laki	10.09	20	Positif Q493R	Neg
135	SNP158	Omicron BA.2	Laki-laki	8.94	23	Positif Q493R	Neg
136	SNP159	Omicron BA.2	Perempuan	25.17	52	Positif Q493R	Neg
137	SNP160	Omicron BA.2	Laki-laki	26.7	64	Positif Q493R	Neg
138	SNP161	Omicron BA.2	Perempuan	22.03	58	Positif Q493R	Neg
139	SNP162	Omicron BA.2	Perempuan	27.98	41	Positif Q493R	Neg
140	SNP038	Omicron BA.2	Laki-laki	21.2	23	Positif Q493R	Neg

**Symptoms**
Mild Symptomatic
Asymptomatic
Mild Symptomatic
Mild Symptomatic
Mild Symptomatic
Mild Symptomatic
Mild Symptomatic
Mild Symptomatic
Mild Symptomatic
Mild Symptomatic
Mild Symptomatic
Mild Symptomatic
Mild Symptomatic
Mild Symptomatic
Mild Symptomatic
Mild Symptomatic
Mild Symptomatic
Mild Symptomatic
Mild Symptomatic
Mild Symptomatic
Mild Symptomatic
Mild Symptomatic
Asymptomatic
Mild Symptomatic
Mild Symptomatic
Mild Symptomatic
Mild Symptomatic
Mild Symptomatic
Mild Symptomatic
Mild Symptomatic
Mild Symptomatic
Mild Symptomatic
Mild Symptomatic
Mild Symptomatic
Mild Symptomatic
Mild Symptomatic
Mild Symptomatic
Mild Symptomatic
Mild Symptomatic
Mild Symptomatic
Mild Symptomatic
Mild Symptomatic
Mild Symptomatic
Mild Symptomatic
Simptomatik Berat
Mild Symptomatic

Mild Symptomatic
Mild Symptomatic
Mild Symptomatic
Mild Symptomatic
Asymptomatic
Mild Symptomatic
Mild Symptomatic
Mild Symptomatic
Mild Symptomatic
Mild Symptomatic
Mild Symptomatic
Mild Symptomatic
Mild Symptomatic
Moderate Symptomatic
Mild Symptomatic
Mild Symptomatic
Mild Symptomatic
Mild Symptomatic
Mild Symptomatic
Mild Symptomatic
Mild Symptomatic
Mild Symptomatic
Mild Symptomatic
Mild Symptomatic
Mild Symptomatic
Mild Symptomatic
Mild Symptomatic
Mild Symptomatic
Mild Symptomatic
Asymptomatic
Mild Symptomatic
Mild Symptomatic
Mild Symptomatic
Mild Symptomatic
Mild Symptomatic
Mild Symptomatic
Mild Symptomatic
Simptomatik berat
Mild Symptomatic
Mild Symptomatic
Mild Symptomatic
Mild Symptomatic
Mild Symptomatic
Mild Symptomatic
Mild Symptomatic
Mild Symptomatic
Mild Symptomatic

Mild Symptomatic
Mild Symptomatic
Mild Symptomatic
Mild Symptomatic
Mild Symptomatic
Mild Symptomatic
Mild Symptomatic
Mild Symptomatic
Mild Symptomatic
Mild Symptomatic
Mild Symptomatic
Mild Symptomatic
Mild Symptomatic
Mild Symptomatic
Mild Symptomatic
Mild Symptomatic
Mild Symptomatic
Mild Symptomatic
Mild Symptomatic
Mild Symptomatic
Mild Symptomatic
Mild Symptomatic
Mild Symptomatic
Mild Symptomatic
Moderate Symptomatic
Mild Symptomatic
Mild Symptomatic
Mild Symptomatic
Mild Symptomatic
Mild Symptomatic
Mild Symptomatic
Mild Symptomatic
Mild Symptomatic
Mild Symptomatic
Mild Symptomatic
Mild Symptomatic
Mild Symptomatic
Mild Symptomatic
Mild Symptomatic
Mild Symptomatic
Mild Symptomatic
Asymptomatic
Asymptomatic
Mild Symptomatic
Mild Symptomatic
Mild Symptomatic
Asymptomatic
